# Severe SARS‐CoV‐2 infection in diabetes was rescued in mice supplemented with metformin and/or αKG, and patients taking metformin, via HIF1α‐IFN axis

**DOI:** 10.1002/ctm2.70275

**Published:** 2025-04-08

**Authors:** Garima Joshi, Garima Verma, Simrandeep Kaur, Ellango Ramasamy, Navya Chauhan, Savita Singh, Pradipta Jana, Zaigham Abbas Rizvi, Pallavi Kshetrapal, Shinjini Bhatnagar, Anil Kumar Pandey, Amit Awasthi, Bhabatosh Das, Prasenjit Guchhait

**Affiliations:** ^1^ Department of Biotechnology Regional Centre for Biotechnology, National Capital Region Biotech Science Cluster Faridabad Haryana India; ^2^ Translational Health Science Technology Institute, National Capital Region Biotech Science Cluster Faridabad Haryana India; ^3^ ESIC Medical College and Hospital Faridabad Haryana India

1

Dear Editor,

Studies report a higher prevalence of COVID‐19 and worse outcomes in diabetic patients, typically 1.5‐3‐fold greater than similar populations without diabetes^1^ and the individuals taking the hypoglycaemic drug metformin have better survival.^2^, ^3^ We investigated the mechanisms for the above reports and observed that the hyperglycaemic mice with either type 1 or type 2 diabetes (T1D/T2D) have elevated SARS‐CoV‐2 infection in the lungs at day‐5 post infection (5DPI, Figure 1 A, B) with aggressive inflammation, indicated by infiltration of immune cells (Figure 1C,D) and accumulation of cytokines TNFα and IL6 (Figure S1C,D) as compared to non‐diabetic counterparts, (T2D mice data in Figure 1L–O, Figure S2C‐D). Both T1D/T2D mice showed decreased levels of interferons in lung tissue lysate (type‐1 and ‐2; IFN‐α/IFN‐β/IFN‐γ, Figure 1H–J/S–U) alongside decreased expression of IFNA1/IFNB1/IFNG1 and IFN‐regulatory factor (IRF)‐3/IRF‐7 genes (Figure‐1E–G/P–R, Figures S1E,F and S2E,F) in the lung tissue and in the spleen (Figures S1I–M and S2I–M) but elevated HIF‐1α in the lungs (Figure 1K,V and Figures S1G and S2G). The supplementation with metformin and/or alpha‐ketoglutarate (αKG, previously used by us^4^, ^5^) significantly reduced viral load, and rescued inflamed lungs and IFNs synthesis (Figure‐1K,V and Figure S1 H/S2 H). Unlike IFNs, the levels of IgG and the neutralization antibody titre against SARS‐CoV‐2 were found unaltered between WT and diabetic (T1D/T2D) mice even after metformin+αKG treatment (Figures S1N–Q and S2N,O). The repetition of above experiment in K‐18 mice and high fat diet induced T2D model of K‐18 showed similar observations (Figure S4A–M). In mechanism, we described that hyperglycaemic microenvironment (25 mM glucose) increased SARS‐CoV‐2 infection (Figure 2A) and decreased IFNA1/IFNB1 expression in the insulin resistant Huh‐7 cells model (Figure S3A,B). The metformin and/or alpha‐ketoglutarate treatment increased the IFN synthesis and decreased viral load (Figure 2A–C). The expression of HIF‐1α inversely corelated with P‐IRF3 suggesting a decreased IFN synthesis in these cells in diabetic microenvironment, and its improvement following metformin and/or αKG treatment (Figure 2D and Figure S3E,F). The inhibition of HIF‐1α function by CAY10585 was found to improve IFN synthesis and inhibiting SARS‐CoV‐2 infection in these cells (Figure 2E and Figure S3G,H). We validated the above mechanism using HIF1α‐depleted (using HIF1A‐shRNA previously used by us^5^) U937 monocytic cells transiently expressing hACE2. HIF1A‐depleted cells have a decreased viral load as compared to cells with control‐shRNA (Figure 2F). Conversely, the IFNA1/IFNB1/IFNG1 transcripts were elevated in HIF1A‐depleted cells than controls (Figure 2G–I). We also observed an elevated P‐IRF3 in the HIF1A‐depleted cells as compared to controls (Figure 2J, Figure S3M,N).

**FIGURE 1:**
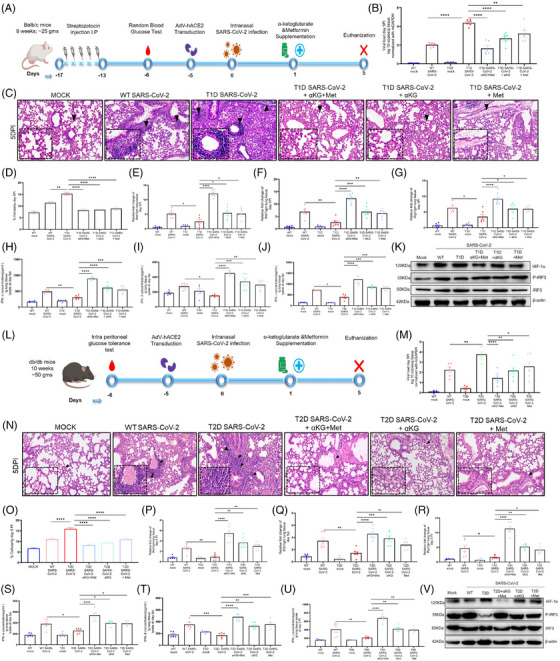
Effect of supplementation with metformin and αKG on SARS‐CoV‐2 infection in mice with type 1 diabetes (T1D) and type 2 diabetes (T2D). (A) Schematic representation of the experimental plan in T1D mice. (B) Supplementation with αKG (10 mg/25 g body weight daily) and metformin (250 mg/kg twice a day) separately and in combination (αKG + metformin) decreased the SARS‐CoV‐2 load in lungs of T1D mice, measured by RT‐PCR at day 5 post‐infection (5DPI) and normalized with mGAPDH. Each dot represents individual mouse. Data are the mean ± SEM. One‐way ANOVA and Sidak's multiple comparison tests were used. (C) H&E staining of the lung tissue at a scale bar of 100 µm. Arrows indicate the infiltration of the cells. (D) Percentage cellularity score was calculated from 12 different fields and different animals from above animals. (E‐G) the mRNA levels of the interferon genes ifna1 (E), ifnb1 (F), and (G) infg1 were quantified in the lung of the infected mice at 5DPI using RT‐PCR. Data are the mean ± SEM and analysed as mentioned above. (H–J) The levels of IFNα, IFNβ and IFNγ were measured using ELISA/CBA‐array from the lung tissue lysate at 5DPI. Data are the mean ± SEM. One‐way ANOVA and Sidak's multiple comparison tests were used. (K) Western blotting from the lung tissue shows decreased expression of HIF‐1α and increased expression of P‐IRF3 in the metformin and αKG supplemented T1D mice infected with SARS‐CoV‐2 (densitometry data Figure S1G,H). (L) Schematic representation of the similar experiments in T2D mice. (M) The SARS‐CoV‐2 viral load was measured in mice lung at 5DPI. Data are the mean ± SEM and Sidak's multiple comparison tests were used. (N) H&E staining of the lung tissue and (O) percentage cellularity score of the same. (P–R) The mRNA levels of the interferon genes ifna1 (P), ifnb1 (Q), and (R) infg1 were quantified in the above mice lung. (S–U) The levels of IFNα, IFNβ and IFNγ were measured from lung tissue of above mice using ELISA/CBA. Data are the mean ± SEM. One‐way ANOVA and Sidak's multiple comparison tests were used. (V) Western blotting from the lung tissue shows less expression of HIF‐1α and increased expression of P‐IRF3 in the metformin and αKG supplemented T2D mice infected with SARS‐CoV‐2 (densitometry data Figure S2G,H). For all Figures, ns = non‐significant, *p < 0.05, **p < 0.01, ***p < 0.001 & **** *p* < 0.0001.

**FIGURE 2:**
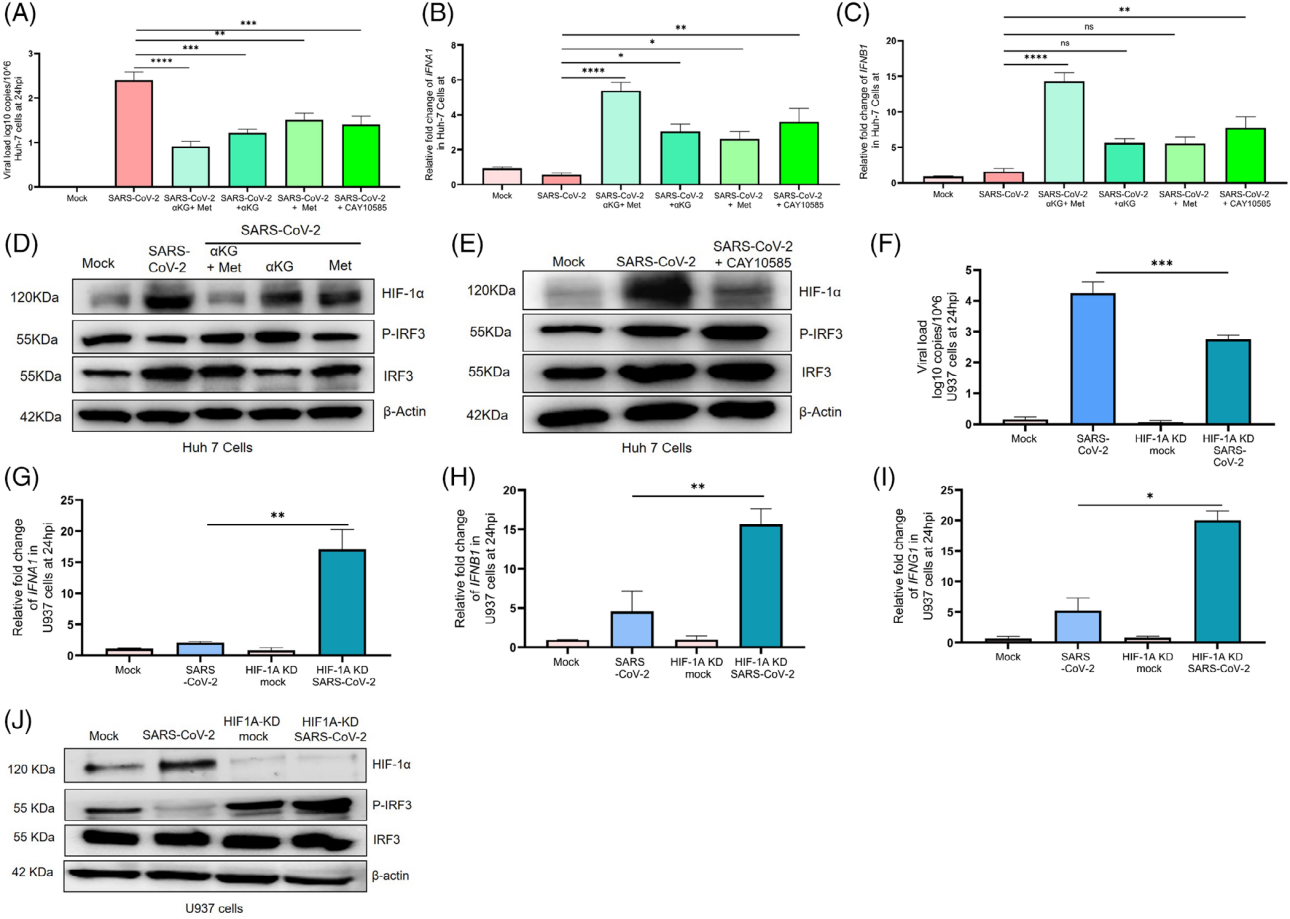
SARS‐CoV‐2 infection and IFN expression in insulin‐resistant Huh7 and U937 cells, and in HIF1A‐KD U937 cells: (A) The viral load of SARS‐CoV‐2 was measured in the insulin resistant Huh7 cells. The cells show decreased viral load in the treatment groups at 24 h‐post infection (hpi). Protocol for making insulin‐resistant cells, is described in methods and Figure S3A,B. The experiment was performed in the presence of 25 mM glucose with octyl‐αKG (1 mM), metformin (5 mM), αKG + metformin, and HIF1α inhibitor CAY10585 (40 µM). Viral load was measured using RT‐PCR normalized with hGAPDH. Data are the mean ± SEM. One‐way ANOVA and Sidak's multiple comparison tests were used. (B, C) mRNA expression of IFNA1 and IFNB1 genes were measured in the above cell pellet. (D) Expression of HIF1α, P‐IRF3, and IRF3 proteins were measured using western blot and normalized with β‐actin at 24 hpi in insulin resistant Huh7 cells under 25 mM glucose. (E) Huh7 cells were infected with SARS‐CoV‐2 in the presence of 25 mM glucose with/without CAY10585 (40 µM), and the expression of HIF1α, P‐IRF3, and IRF3 was quantified by western blot and normalized with β‐actin. SARS‐CoV‐2 infection in HIF‐1A KD U937 cells. To further investigate the correlation between HIF1α and IFN axes, HIF1A‐knockdown cells were used. (F) HIF1A‐KD cells were infected with SARS‐CoV‐2 at 0.1 MOI, and after 24 h, the viral load was measured from the cell pallets using RT‐PCR. (G–I) mRNA expression of interferon genes (G) IFNA1, (H) IFNB1, and (I) IFNG1 was measured using RT‐PCR. Data represented as median and IQR, *n* = 3. One‐way ANOVA and Kruskal Wallis Dunn's multiple comparison tests were used. (K) Expression of HIF1α, P‐IRF3, and IRF3 proteins were measured using western blot and normalized with β‐actin. For all Figures, ns = non‐significant, *p < 0.05, **p < 0.01, ***p < 0.001, and **** *p* < 0.0001. HIF1A‐KD cells were generated using HIF1A shRNA and confirmed using western blot. ACE2 was expressed transiently in the HIF1A‐KD U937 cells using a hACE2 adenovirus construct (Figure S6F–I).

Hyperglycaemia has been associated with severe COVID‐19 in patients with T1D/T2D.^6^, ^7^ Elevated glucose increases COVID‐19 via HIF‐1α/glycolysis pathway.^6^ In‐vitro studies have described that hyperglycaemia increased ACE2 expression in pancreas and promotes SARS‐CoV‐2 infection.^7^ Our data have described an increased SARS‐CoV‐2 infection in the lungs of K‐18 hACE2 mice with T2D (Figure S4). Our data also described the suppression of HIF‐1α, a negative regulator of the IRF‐IFN axis, by metformin in‐vitro and in‐vivo. HIF‐1α negatively regulates IFN response. Other studies have described the correlation of low IFN levels with higher COVID‐19 infection. It has been described that the hypoxic monocytes in COVID‐19 patients produce less IFNα via activation of HMGB1. HIF‐1α acted as a direct suppressor of IRF5/IRF3 signalling.^8^ Recently, we described that αKG supplementation rescued the COVID‐19 pathogenesis in mice by augmenting prolyl‐hydroxylase‐2 (PHD2) activity, in‐turn suppressing HIF‐1α/P‐AKT.^4^, ^5^

We further validated the above mechanisms in patients’ samples. Samples were collected between June 2020 and April 2021 when Wuhan, alpha (B.1.1.7) and delta (B.1.617.2) strains were prominent in India. The differential gene analysis of PBMCs described that the diabetic COVID‐19 patients taking a higher dose of metformin (1000 mg/day) had upregulation of genes INFAR2 and IFNGR1, and pathways like interferon and cytokines in conjunction with lesser symptoms (Figure 3G, Table S1) than the counterparts taking lower dose (500 mg/day) of the drug, depicted in volcano‐plot (Figure 3A), network analysis (Figure 3B), diverging bar chat (Figure 3C) and Sankey and dot‐plot analysis (Figure 3D). Importantly, the above patients with high dose of metformin showed elevated levels of IFN‐γ in plasma as compared to counterparts with low dose of metformin (Figure‐3E). Furthermore, in‐vitro stimulation of CD3‐T cells from COVID‐19 patients with SARS‐CoV‐2‐RBD peptides showed an elevated IFN‐γ expression in patients taking higher metformin (Figure 3F). Further, data from diabetic COVID‐19 patients taking metformin revealed upregulation of genes including IFNG, IFNGR1, IFNB1, IFNA5, IRF7 and IRF3 as compared to COVID‐19 patients without diabetes and without metformin (Figure 4A–C), indicating an anti‐viral role of this pleiotropic drug. We validated the above findings using RT‐PCR analysis. Data from diabetic COVID‐19 patients taking metformin, showed higher expression of IFNA1/IFNB1/IFNG1/HIF1A and IRF‐3 genes than non‐diabetic COVID‐19 patients without metformin (Figure 4D–H). The levels of IgG antibodies in plasma did not show any difference between diabetic COVID‐19 patients with metformin and non‐diabetic counterparts without metformin (Figure 4I,J) However, the neutralizing antibodies against SARS‐CoV‐2 were higher in metformin‐treated patients than non‐metformin counterparts (Figure 4K,L), indicating better adaptive immune responses of this drug against COVID‐19. Several studies have described that metformin can directly impact leucocytes to suppress inflammation. Metformin significantly increased serum influenza vaccine‐specific antibodies and Th‐1 cell in diabetic individuals.^9^, ^10^ Likewise, we also observed an improved humoral immune response in COVID‐19 patients after metformin treatment. However, a further detailed study may explain the mechanisms of our above observations. In mechanism, we observed that PBMCs of the healthy individuals treated with metformin and exposed to hypoxia (5% O_2_) hypoxia for 1 h showed decreased expression of HIF‐1α (Figure 4M–P), indicating an inverse correlation between HIF‐1α and IFN axes.

**FIGURE 3:**
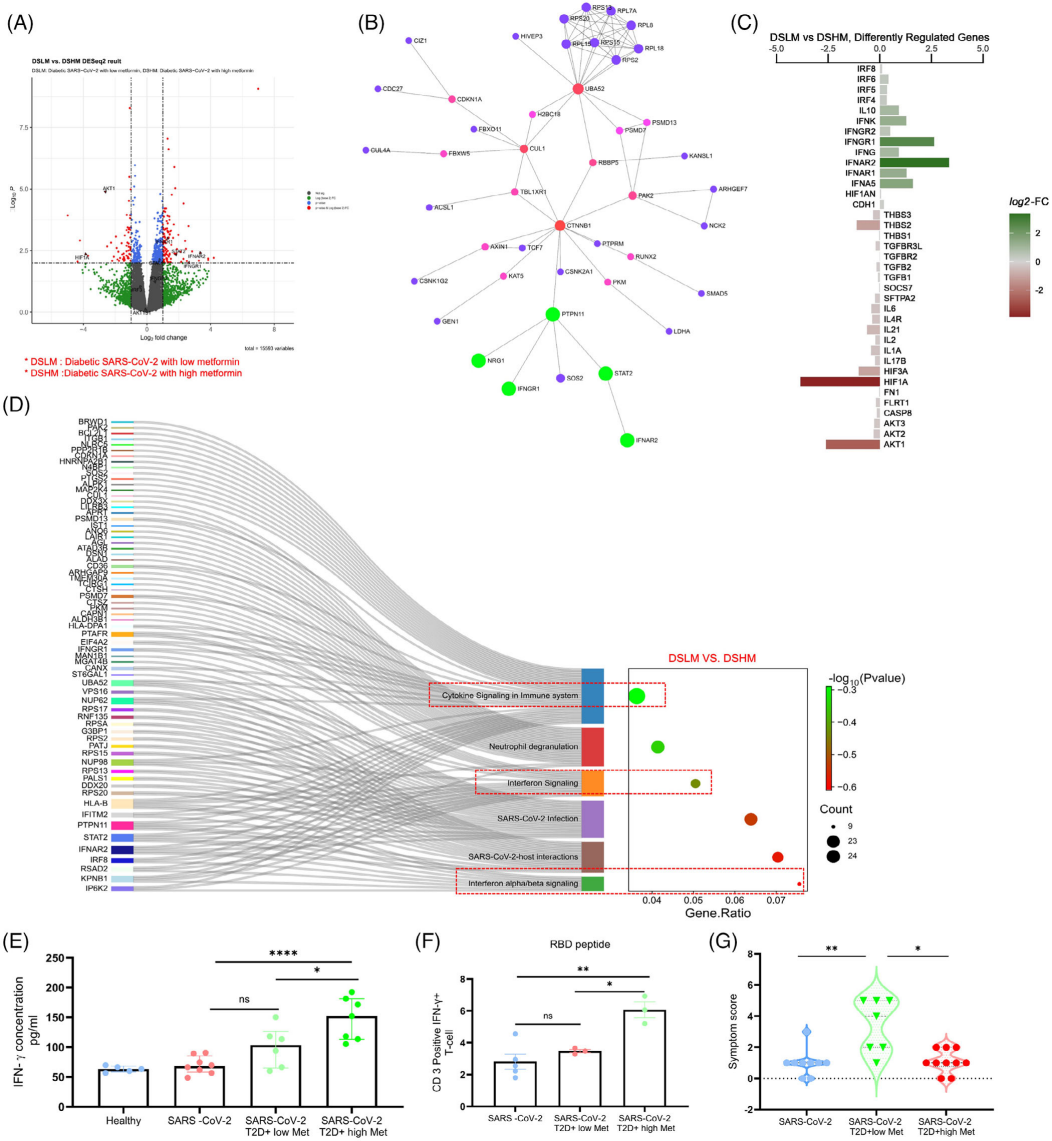
Estimating IFNs and related genes in PBMCs of COVID‐19 infected T2D patients taking lower/higher doses of metformin, and COVID‐19 patients without diabetes, using a next‐generation sequencing platform. The differential gene analysis of whole RNA sequencing data from PBMCs of diabetic COVID‐19 patients taking higher (1000 mg daily, *n* = 5) vs lower (500 mg daily, *n* = 5) doses of metformin, is illustrated in (A) volcano plots, (B) Network, and (C) diverging bar chat (D) Sankey and dot plot analysis of above genes highlighting the interferon and cytokine pathways. (E) The plasma level of IFNγ was measured in the above patients as well as non‐diabetic COVID‐19 patients, and healthy individuals (as control reference), using CBA assay. (F) PBMCs from the above individuals were stimulated with SARS‐CoV‐2 RBD Peptide. The IFN‐γ positive CD3+T cells were measured using flow cytometry. Data are the mean ± SEM. One‐way ANOVA and Sidak's multiple comparison tests were used. (G) The symptom score of T2D patients with COVID‐19 taking high or lower dose of metformin, and COVID‐19 patients without diabetes. Unpaired student t‐test is used. (The table for symptom score is mentioned in supplementary Table S1).

**FIGURE 4:**
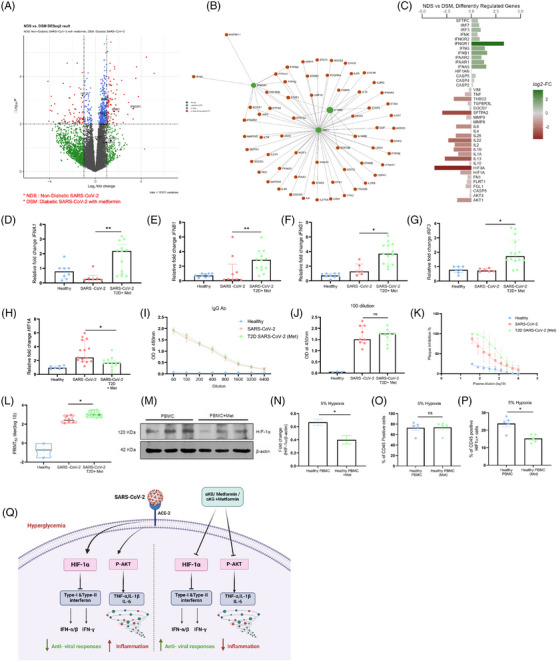
Measuring IFNs and IgG of COVID‐19 patients with diabetes taking metformin, versus COVID‐19 patients without diabetes/metformin‐treatment. The differential gene analysis of whole RNA sequencing data from PBMCs of above group of patients (*n* = 10 each) is illustrated in (A) Volcano plot and (B) Network analysis of the differential genes. (C) diverging bar chat, (D–H) The mRNA expression of interferon genes IFNA1 (D), IFNB1 (E), IFNG1 (F), IRF3 (G) & HIF‐1A (H) in the PBMCs from above individuals was measured using RT‐PCR. Each dot represents an individual sample. Data represented as median and IQR. One‐way ANOVA and Kruskal Wallis Dunn's multiple comparison tests were used. (I) Plasma IgG was measured between groups. Data are the mean ± SEM. One‐way ANOVA and Sidak's multiple comparison tests were used. (J) Absorbance at sera dilution 100 data is represented as median and IQR and calculated as Unpaired student *t*‐test (Mann–Whitney test). (K,L) The neutralization antibody against SARS‐CoV‐2 was measured at increasing sera dilution using a PRNT50 assay between COVID‐19 without (*n* = 6) versus with metformin (*n* = 15). (M,N) The PBMCs of healthy individuals were treated with metformin and exposed to 5% hypoxia for 1 h and a western blot was performed to measure HIF‐1α. Densitometry data of the same from triplicate blots, *n* = 3. (O,P) Percentage CD45 positive cells and HIF‐1α positive CD45 positive cells were measured from the above experiment using flow cytometry. Data represented as median and IQR. Unpaired student *t*‐test (Mann–Whitney test). For all Figures L, M, and N. ns = non‐significant, *p < 0.05, **p < 0.01. (Q) Schematic of the study. Supplementation with metformin and αKG rescues the IFNs and decreases viral infection in diabetic mice. T2D patients taking metformin have elevated IFN‐mediated antiviral response. Metformin suppressed the inhibitory effects of HIF‐1α on IFNs and improved IFN synthesis.

Studies together describe unique effect of metformin along with αKG in improving the IFN synthesis by suppressing HIF‐1α signalling. Studies thus suggest the usage of metformin as the hypoglycaemic drug for treating diabetes on the clinical course of COVID‐19. Studies also suggest that metformin in combination with αKG potentially inhibits COVID‐19 (depicted in schematic Figure 4Q). This may be applicable to other viral infections that increase HIF‐1α signalling and alleviate IFNs.

However, caveats of our study include the mechanism of HIF‐1α‐mediated alleviation of IFNs and metformin‐mediated rescue in‐vivo in patients. Investigating the above mechanism between diabetic COVID‐19 patients without metformin or other hypoglycaemic drugs versus counterparts taking metformin. Unfortunately, samples for the earlier category are not available at our national repository.

## AUTHOR CONTRIBUTIONS

Garima Joshi has designed and performed all experiments including in vivo experiments, analysed RNA sequencing data and wrote part of the original draft of manuscript. Garima Verma has performed experiments using mice and human blood samples, and wrote part of the original draft and edited the final draft of the manuscript. Simrandeep Kaur has performed in vitro and in vivo experiments. Navya Chauhan has performed PCR assays and wrote part of the original draft of manuscript. Shinjini Bhatnagar and Anil Kumar Pandey were project coordinators of the DBT India Consortium. Savita Singh and Pallavi Kshetrapal have stratified the participant samples and clinical information, and have performed clinical correlation analysis of the patients’ data. Zaigham Abbas Rizvi and Amit Awasthi performed the K‐18 mice infection study and analysed data. Pradipta Jana and Bhabatosh Das have generated the RNA sequencing data, and Ellango Ramasamy and Bhabatosh Das analysed the data. Prasenjit Guchhait has conceptualized and supervised whole study and analysed data and wrote the original draft of the manuscript. All authors read, edited and approved the final manuscript.

## CONFLICT OF INTEREST STATEMENT

The authors declare no conflicts of interest.

## FUNDING INFORMATION

The work was supported by the grants: BT/CS0094 from the Department of Biotechnology, Govt. of India; and ICMR/14/1923 from the Indian Council of Medical Research, Govt. of India to Prasenjit Guchhait.

## ETHICS STATEMENT

Approval was also obtained from the Institutional Biosafety Committee (IBSC; ref. no. RCB/IBSC/22‐23/433) of RCB. All the Covid‐19‐related in vitro and animal experiments were performed in the BSL3 facility of RCB. Animal experiment protocol was approved by the Institutional Animal Ethics Committee (IAEC) of Regional Centre for Biotechnology (RCB) (ref. no. RCB/IAEC/2022/116)

## Supporting information



Supporting Information

## Data Availability

The datasets presented in this study can be found in online repositories, GEO, NCBI, SRA, reference number SUB14556384.
