# Supplementary material for: Severe SARS‐CoV‐2 infection in diabetes was rescued in mice supplemented with metformin and/or αKG, and patients taking metformin, via HIF1α‐IFN axis
Source: Clin Transl Med. 2025 Apr 8;15(4):e70275. doi: 10.1002/ctm2.70275 (PMC11978732; doi:10.1002/ctm2.70275)
Supplement: Supplementary file 1 — Supporting Information [file CTM2-15-e70275-s001.pdf]

Severe SARS-CoV-2 infection in diabetes was rescued in mice supplemented with metformin and/or αKG, and patients taking metformin, via HIF1α-IFN axis.

Garima Joshi et al. *Correspondence to Prasenjit Guchhait at prasenjit@rcb.res.in

Supplementary figures:

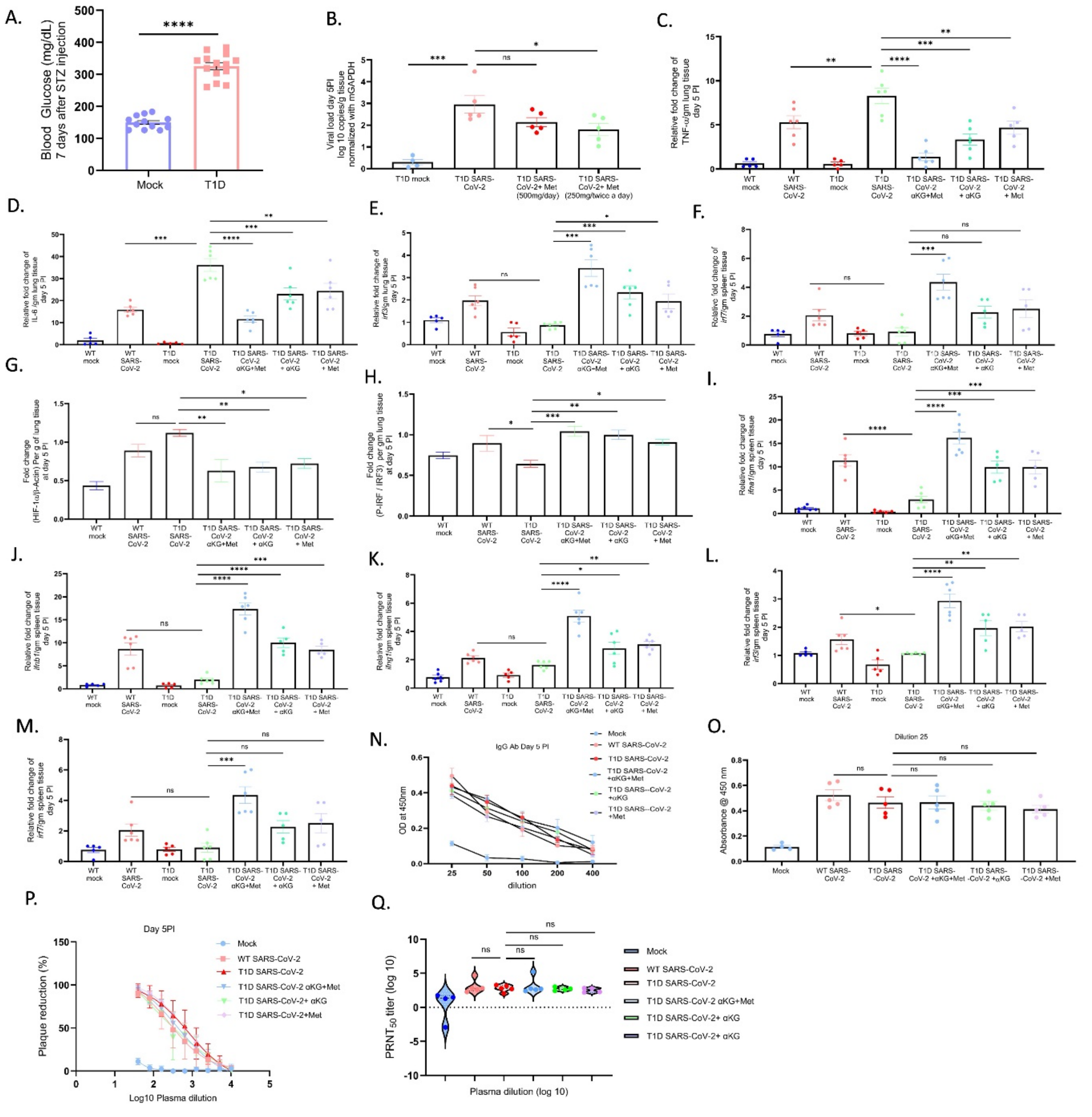

Figure S1. Extension of Fig-1 describes 5DPI data for T1D mice. (**A**) Random blood glucose levels in T1D and WT-healthy mice. Data are the mean ± SEM, and unpaired t-test was used for analysis. **(B)** Supplementation with a dose of metformin 250mg/kg twice a day reduces the viral load in the lung tissue as compared to a single dose 500mg/kg, measured using RT-PCR

and normalized with mGAPDH. Data are the mean ± SEM. One-way ANOVA and Sidak's multiple comparison test were used. **(C-D)** The mRNAs of pro-inflammatory cytokines **(C)** TNF-α and **(D)** IL-6 were quantified in the lung tissue of infected mice using RT-PCR. **(E, F)** The mRNAs of interferon regulatory factors *irf3* (E) and *irf7* (F) were measured from the lung tissue, and normalized with mGAPDH. **(G, H)** Densitometry data from 4 different animals were normalized with β-actin for HIF-1α and IRF3 for P-IRF3 from Fig-1K. **(I-M)** The mRNA levels of the interferons: *ifna1*(I)*, ifnb1*(J)*,* and *ifng1*(K), and interferon regulatory genes *irf3* (L), and *irf7* (M) were measured from the spleen tissues, and normalized with mGAPDH. Data are the mean ± SEM. One-way ANOVA and Sidak's multiple comparison test were used. **(N)** The levels of IgG were measured at increasing sera dilution in the plasma of the infected mice. **(O)** Absorbance at sera dilution 25, at 5DPI. **(P, Q)** The neutralization antibody was measured at increasing sera dilution using a $PRNT_{50}$ assay showing no difference between the above groups. Data represented as mean ± SEM were calculated as mentioned above. For all Figures, ns= non-significant, **P<0.01, ***P <0.001 & **** P < 0.0001.

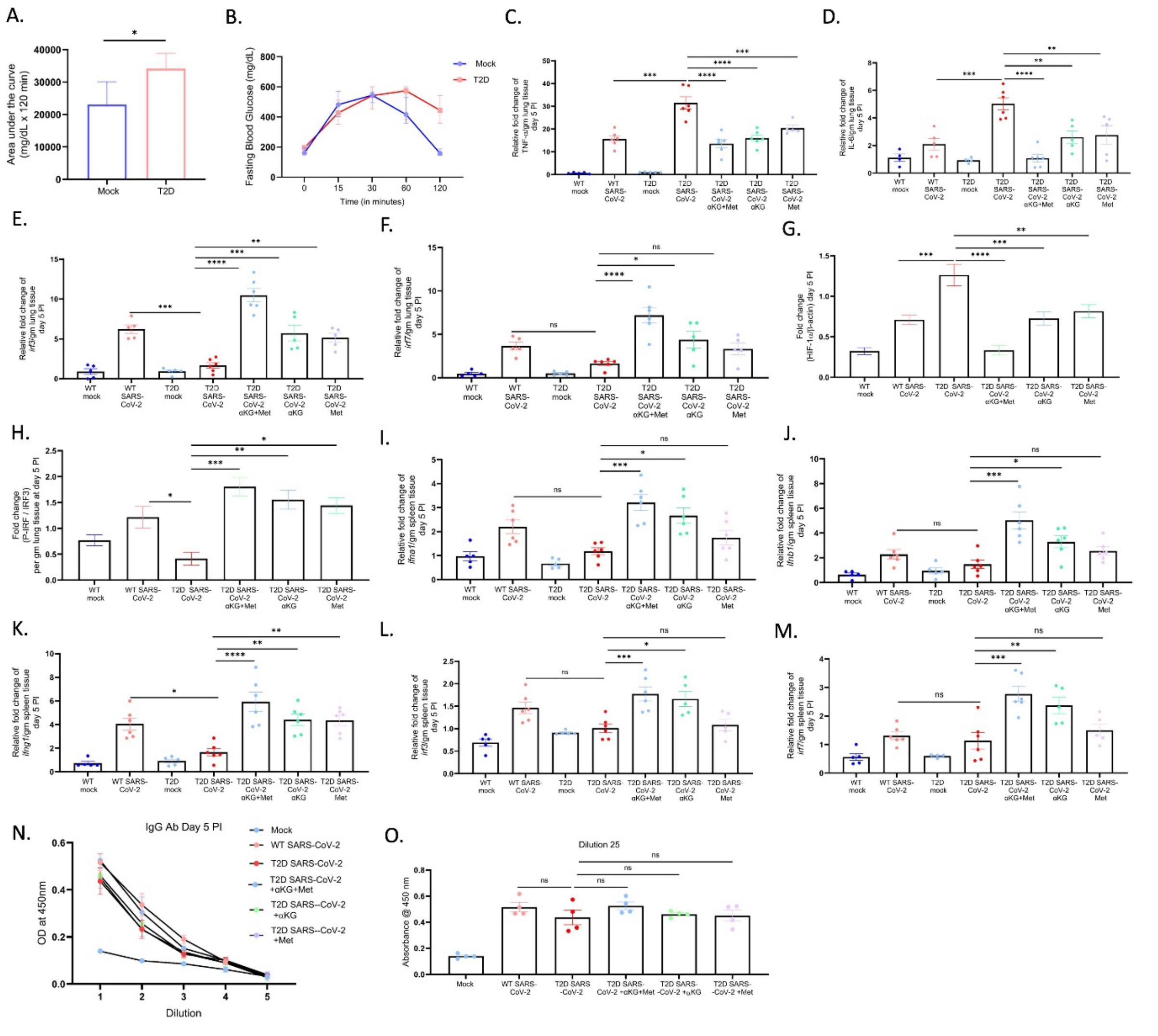

Figure S2. Extension of Figure 1 describes 5DPI data for T2D mice. (**A, B)** The graph depicts the area under the curve for fasting blood glucose levels in type 2 diabetes (T2D) (db/db) mice and WT-healthy controls. Data are the mean ± SEM unpaired t-test. **(C-D)** Expression of the

pro-inflammatory cytokines TNF-α and IL-6 genes was measured in the lungs of infected mice using RT-PCR. Each dot represents one mouse value. Data are the mean ± SEM. One-way ANOVA and Sidak's multiple comparison test were used. **(E, F)** The mRNAs of interferon regulatory factors *irf3* (E) and *irf7* (F) were measured from the lung tissue using RT-PCR, and normalized with mGAPDH. **(G, H)** Densitometry data from 4 different animals were normalized with β-actin for HIF-1α and IRF3 for P-IRF3 from Fig-1V. Data are the mean ± SEM. One-way ANOVA and Sidak's multiple comparison test were used. **(I-M)** The mRNA levels of the interferons: *ifna1*(I), *ifnb1*(J), and *ifng1*(K), and interferon regulatory genes *irf3* (L) and *irf7* (M) were measured from the spleen tissues and normalized with mGAPDH. Data are represented as mean ± SEM and comparison test were used as mentioned above. **(N)** The levels of IgG Ab at increasing sera dilution in the plasma of the infected mice. **(O)** Absorbance at sera dilution 25 at 5DPI. Data represented as mean ± SEM were calculated as mentioned above. For all Figures, ns= non-significant, **P<0.01, ***P <0.001 & **** P < 0.0001.

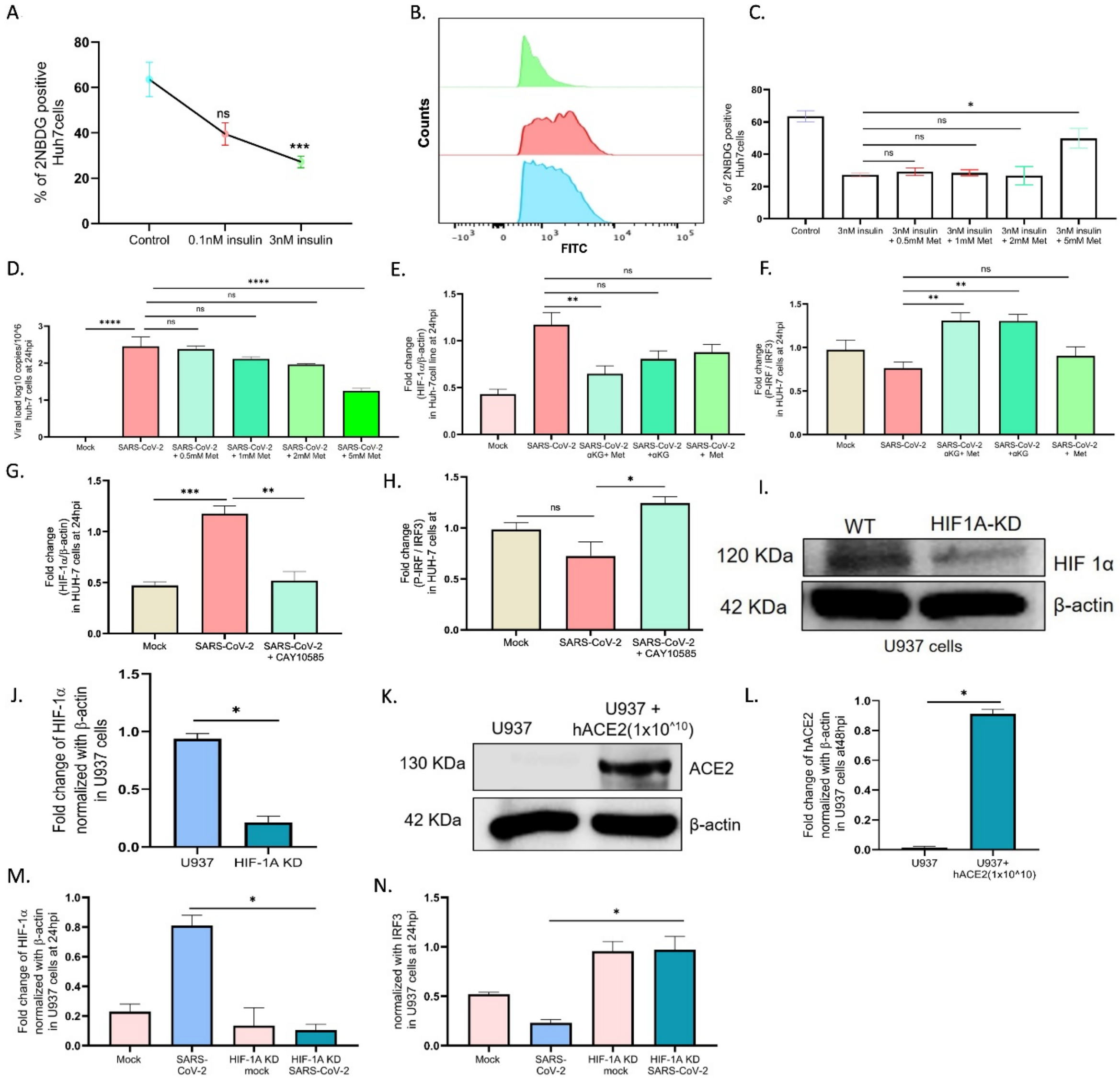

Figure S3. Extension of Figure 2 describes in-vitro data. **(A)** Glucose uptake assay showing the percentage of 2NBDG positive cells upon treatment with insulin 0.1nm and 3nm in Huh-7 cell line at 48hr post treatment. **(B)** Huh7 cells were assessed for glucose uptake of fluorescent glucose analog 2-NBDG after the treatment of insulin for two days and results are shown as

histogram where blue colour indicates control group, red and green indicates 0.1nm and 3nm insulin treatment. **(C)** Glucose uptake assay showing the percentage of 2NBDG positive cells upon treatment with different concentration of metformin in insulin resistance Huh-7 cell line. Data are the mean ± SEM. One-way ANOVA and Sidak's multiple comparison test were used. **(D)** The viral load of SARS-CoV-2 was measured using RT-PCR, with different metformin concentration at 24hrpi. Data are the mean ± SEM. One-way ANOVA and Sidak's multiple comparison test were used. **(E-H)** Densitometry analysis of protein bands of HIF-1α, and P-IRF3 from main Figures 2D &E. **(I-J)** HIF1A-knockdown U937 cells were generated using HIF1A shRNA and confirmed using a western blot. (**K-L)** ACE2 was expressed transiently in the HIF1A-KD U937 cells using a human ACE2 adenovirus construct. **(M-N)** Densitometry for HIF-1α, P-IRF3 from main Figure 2J. Data are as median and IQR, n=3. One-way ANOVA and Kruskal Wallis Dunn's multiple comparison test were used. For all Figures, ns= non-significant, *P<0.05, **P<0.01, ***P <0.001.

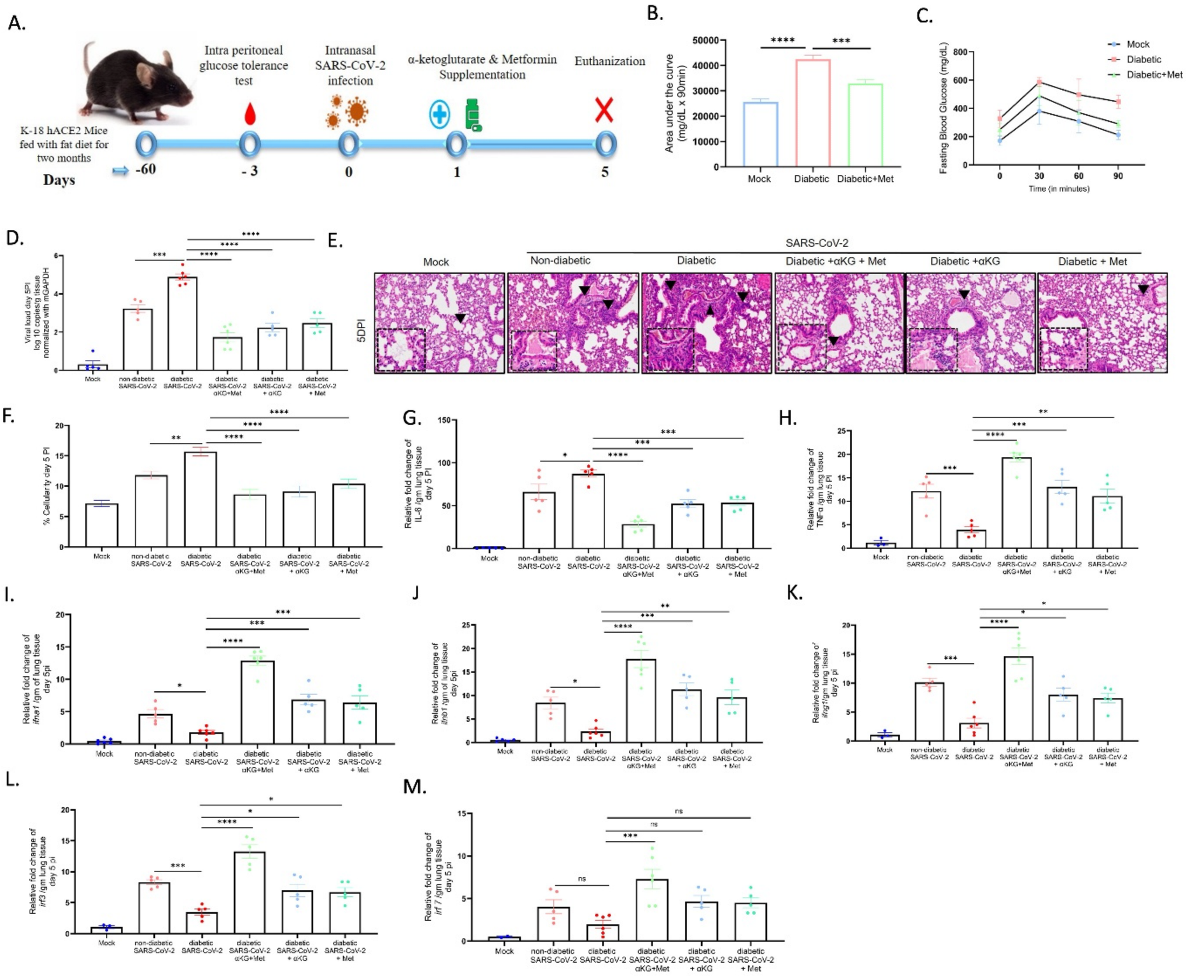

Figure S4. (A) Schematic representation of the experimental plan in high-fat diet induced diabetes in K-18 hACE2 transgenic mice, and also SARS-CoV-2 infection and experiment plan. **(B, C)** The graph depicts the area under the curve for fasting blood glucose levels in K-18 hACE2 transgenic mice with and without metformin treatment (250mg/kg twice a day for five days). **(D)** Supplementation with αKG and metformin decrease the viral load in SARS-CoV-2 infected diabetic K-18 hACE2 transgenic mice lungs measured with RT-PCR at day five post-infection (5DPI) and normalized with mGAPDH. Each dot represents one mouse value.

(E) H&E staining of the lung tissue at a scale bar of 100µm. Arrows indicate the infiltration of the cells. **(F)** Percentage cellularity score was calculated from 10 different fields and different animals. **(G, H)** The mRNA levels of pro-inflammatory cytokines IL-6 (G) and TNF-α (H) were measured from the lung tissue using RT-PCR. (**I-M)** the mRNA levels of the interferon genes *ifna1*(I), *ifnb1* (J), and (K) *infg1* and interferon regulatory genes *irf3* (L), and (M) *IRF7* were quantified in the lung of the infected mice using RT-PCR. Data are consistently represented as mean ± SEM One-way ANOVA and Sidak's multiple comparison tests were used. For all Figures, ns= non-significant, *P<0.05, **P<0.01, ***P <0.001 and ****P <0.0001.

Supplementary Tables:

Table S1: Clinical information of Covid-19 patients. PBMCs were used for RNA sequencing analysis as well as for other assays like flow cytometry and RT-PCR:

S.No.	Pateint ID	Patient type	COVID-19	Fever	Cough	sore throat	breathlessness	Oxygen	Symptom score	Anti-diabetic drugs	date of hospitalization
1	C-09-2703-3	Non-diabetic	Positive	Yes	No	No	No	No	1	_	02-12-2020
2	C-09-2724-3	Non-diabetic	Positive	No	No	Yes	No	No	1	_	03-12-2020
3	C-09-3082-3	Non-diabetic	Positive	No	No	No	No	No	0	_	02-12-2020
4	C-09-3820-3	Non-diabetic	Positive	No	Yes	No	No	No	1	_	27-04-2021
5	C-09-3523-3	Non-diabetic	Positive	Yes	No	No	No	No	1	_	28-03-2021
6	C-09-3824-3	Non-diabetic	Positive	Yes	No	No	No	No	1	_	26-04-2021
7	C-09-1565	Diabetic	Positive	Yes	Yes	Yes	Yes	No	4	Metformin(500mg)	11-07-2020
8	C-09-2274	Diabetic	Positive	No	No	Yes	No	No	1	Metformin(500mg)	18-10-2020
9	C-09-2329	Diabetic	Positive	Yes	No	Yes	No	No	2	Metformin(500mg)	25-10-2020
10	C-13-0536	Diabetic	Positive	Yes	Yes	Yes	Yes	Yes	5	Metformin(500mg)	15-06-2020
11	C-09-1990	Diabetic	Positive	Yes	Yes	Yes	Yes	Yes	5	Metformin(500mg)	19-09-2020
12	C-13-1468	Diabetic	Positive	Yes	No	Yes	No	No	2	Metformin(500mg)	01-10-2020
13	C-13-1772	Diabetic	Positive	Yes	Yes	Yes	Yes	Yes	5	Metformin(500mg)	12-11-2020
14	C-13-0593	Diabetic	Positive	Yes	No	No	Yes	No	2	Metformin(1000mg)	20-06-2020
15	C-13-1020	Diabetic	Positive	Yes	No	No	No	No	1	Metformin(1000mg)	28-07-2020
16	C-13-1192	Diabetic	Positive	Yes	No	No	No	No	1	Metformin(1000mg)	23-08-2020
17	C-13-1523	Diabetic	Positive	Yes	No	No	No	No	1	Metformin(1000mg)	10-10-2020
18	C-13-1767	Diabetic	Positive	Yes	No	No	No	No	1	Metformin(1000mg)	13-11-2020
19	C-13-1189	Diabetic	Positive	Yes	No	No	No	No	1	Metformin(1000mg)	21-08-2020
20	C-13-1495	Diabetic	Positive	No	Yes	Yes	No	No	2	Metformin(1000mg)	07-10-2020
21	C-13-1588	Diabetic	Positive	No	No	No	No	No	0	Metformin(1000mg)	21-10-2020
22	C-13-1668	Diabetic	Positive	Yes	No	Yes	No	No	2	Metformin(1000mg)	31-10-2020
23	C-13-1735	Diabetic	Positive	No	No	No	No	No	0	Metformin(1000mg)	08-11-2020

Table S2: Primer list:

**Mouse (Gene)**	**Forward primer(5'-3')**	**Reverse primer(5'-3')**
GAPDH	ACCACAGTCCATGCCATCAC	TCCACCACCCTGTTGCTGTA
Ifna1	ATGGCTAGRCTCTGTGCTTTCCT	AGGCTCTCCAGAYTTCTGCTCTG
Ifnb1	GCAGTGGGTGGAATGAGACTATTG	TTCTGAGGCATCAACTGACAGGTC
Ifng1	GGCCATCAGCAACATAAGCGT	TGGGTTGTTGACCTCAAACTTGGC
Irf3	GGCTTGTGATGGTCAAGGTT	ATGTCCTCCACCAAGTCCTG
Irf7	ACAGCACAGGGCGTTTTATC	GAGCCCAGCATTTTCTCTTG
TNFA	CTGAACTTCGGGGTGATCGGT	ACGTGGGCTAGAGGCTTGTCA
IL-6	AGTTGCCTTCTTGGGACTAC	TCCACGATTTCCCAGAGAAC

**Human (Gene)**		
GAPDH	GCCACATCGCTCAGACACCAT	ACCAGGCGCCCAATACG
IFNA1	TGGGCTGTGATCTGCCTCAAAC	CAGCCTTTGGAACTGGTTGCC
IFNB1	TGGCAATTGAATGGGAGGCT	TCATAGATGGTCACTGCGGC
IFNG1	GAATGGCCAACGCAAAGCAA	TCCTTGTTCGCTTCCCTGTTT
IRF3	ATTTCCGCTCTGCCCTCAAC	GAGGTGTCTGGCTGGGAAAA
HIF1A	GAACGTCGAAAAGAAAAGTCTCG	CCTTATCAAGATGCGAACTCACA
SARS-CoV-2		
Spike	GTGTCATGGTGGCGAATAAG	TCGTTGAACCAGGGACAAGG

Table S3: Antibody list:

Sl no.	antibody	manufacture	clone	catalog no.
1.	*P-IRF 3*	Cell Signalling Technology	4D4G	4947
2.	*IRF 3*	Cell Signalling Technology	D83B9	4302
3.	*HIF 1α*	Cell Signalling Technology	D2U3T	14179S
4.	*β- ACTIN*	Cell Signalling Technology	13E5	4970S
5.	*ACE-2*	Cell Signalling Technology		9248ST
6.	Goat anti-rabbit HRP conjugated	Cell Signalling Technology		7074
7.	Goat anti-mouse HRP conjugated	Cell Signalling Technology		7076
8.	CD45 Anti-mouse	BioLegend	104	109814
9.	CD41 anti-mouse	BioLegend	EBioMWReg30	4338315
10.	CD3 Anti-mouse	BioLegend	OKT3	317318
11.	IFN-Gamma anti-human	BioLegend	4S.B3	502516
12.	2-NBDG	Thermo fisher Scientific		N13195

Methods

Ethics

Small animals: Animal experiment protocol was approved by the Institutional Animal Ethics Committee (IAEC) of Regional Centre for Biotechnology (RCB) (ref. no.

RCB/IAEC/2022/116) and experiments using Balb/c mouse strain (RRID: IMSR_JAX_000651) and C57B6 and, db/db/ were conducted within the guideline of IAEC in the Small Animal Facility (SAF) of our institute.

Human subjects: Blood samples from patients and healthy participants were collected under the approved protocol from the Institutional Ethics Committee (RCB-BBB-IEC-H-34/35). Plasma and PBMCs of Covid-19 patients were collected from the National Covid-19 Biorepository by Govt. of India at the Translational Health Science and Technology Institute, Faridabad, India. Blood samples were collected 6-10 weeks post-infection. Plasma and PBMCs were isolated using the Ficoll-Paque density gradient centrifugation method and subsequently cryopreserved in liquid nitrogen for further use at the biorepository. Samples were collected between June 2020 and April 2021 when the original Wuhan strain, alpha strain (B.1.1.7), and delta strain (B.1.617.2) were prominent in India. Unfortunately, we do not have patients' data with particular strain.

Biosafety: Approval was also obtained from the Institutional Biosafety Committee (IBSC; ref. no. RCB/IBSC/22-23/433) of RCB. All the Covid-19-related in vitro and animal experiments were performed in the BSL3 facility of RCB.

Cells and virus

To investigate the mechanism insights, we performed the in vitro studies using cell lines including the human liver cell line Huh 7, monkey kidney cell line Vero E6, and human monocytic cell line U937[all obtained from ATCC, USA). Previously we used these cell lines for our SARS-CoV-2 studies[4,5]. Cells were cultured at 37°C with 5% CO_2 and 70-80% humidity in DMEM and RPMI (Invitrogen and Sigma Aldrich)] media, supplemented with 10% FBS (Gibco) and 1% penicillin-streptomycin. These cells, confirmed to be free of mycoplasma contamination, were used for the subsequent experiments. The SARS-CoV-2 strain USA-WA-1/2020 (obtained from the University of Texas Medical Branch, USA) was utilized at an MOI of ~0.1, as detailed in our previous work[4,5].

Insulin resistance in Huh7 cells:

The insulin resistance was induced via hyperinsulinemia. Huh7 cells were exposed with two different dose of insulin 0.1nM and 3nM for 48hrs and media was replenished every 12hr.

After 48 hr of treatment to washout the insulin, cells were washed with DMEM for seven times, including three quick washes followed by three 5 min. wash and a long 20 min wash with DMEM at 37°C. Subsequently an acute insulin response was elected by treating the cells with 120nm for 10 minutes in DMEM with 1.25% fatty acid free BSA at 37°C with 5% CO_2 in a humidified incubator[13]. To check the insulin resistance glucose uptake assay was performed using a florescent labelled glucose analogue 2-NBDG. After 48 hr insulin treatment followed by insulin shock the cells were then stained with 2-NBDG and propodeum iodide for 20 min. cells were then washed and analysed using a BD FACS Verse flow cytometer (BD Biosciences), and the data were processed with FlowJo software (FlowJo LLC, Oregon).

Virus preparation

The SARS-CoV-2 strain was propagated in Vero E6 cells cultured in DMEM supplemented with 10% FBS and penicillin-streptomycin. Cells were infected with SARS-CoV-2 at MOI of ~0.1 for 1 hr at 37°C and 5% CO_2. Post-infection, the medium was replaced with DMEM containing 2% FBS. Cells were then incubated at 37°C and 5% CO_2 for 48 hrs. The supernatant was collected, centrifuged at 2000 rpm for 10 minutes at 4°C, and filtered through a 0.2 µm

membrane. The purified supernatant was aliquoted and stored at -80°C. Infectious particles were quantified as plaque-forming units (PFU)[4,5].

Plaque forming assay

The SARS-CoV-2 virus stock was serially diluted and used to infect Vero E6 cells for 1 hour. Following infection, cells were overlaid with 2% CMC and incubated at 37°C with 5% CO2 for 2 days. After removing the CMC, cells were washed with PBS and fixed with 4% paraformaldehyde for 20 minutes. Cells were then stained with 1% crystal violet for 10 minutes and washed with tap water. Plaques were counted to determine the viral titer[5].

Mouse infection

To investigate the mechanism of severe pathogenesis of Covid-19 in diabetes, we used streptozotocin-induced type 1 diabetes (T1D) mice, db/db type 2 diabetes (T2D) mice models, and K-18 h ACE2 transgenic mice previously used by us[12]. Balb/c, C57BL/6, and db/db mice were obtained from the Jackson Laboratory, USA, and maintained at the Experimental Animal Facility (EAF), RCB, Faridabad, India and K-18 hACE2 transgenic mice were obtained from Dr. Amit Awasthi, THSTI, Faridabad, India. Balb/c mice aged 6-8 weeks were used to develop T1D model. Mice were induced with five consecutive intraperitoneal doses of streptozotocin (50 mg/Kg), and blood glucose levels were measured after one week using a glucometer. The db/db and wild-type C57BL/6 mice aged 10-12 weeks were used as T2D model. The K-18 h ACE2 transgenic mice 10-12week old were fed with high fat diet (Rodent purified diet W/60% energy from Fat diet, Research Diet INC. Cat no.D12492) for two month and blood glucose levels were measured using glucose tolerance test. The Balb/c, C57BL/6, and db/db mice were inoculated intranasal with 25×10^5 PFU/ml of human ACE-2 adenovirus vector for transient hACE-2 expression in the respiratory tract (schematic Fig.1A and Fig.1L). Five days later, mice were infected intranasal with 1 × 10^6 PFU/ml of SARS-CoV-2 Wuhan strain (reference USA-WA-1/2020). Mice were treated daily with αKG (10mg/25g body weight)[12] once a day and 250mg/kg metformin twice[14] until 5 days post-infection (DPI). Animals were sacrificed, and lung and spleen tissues were collected for following assays.

Real-Time PCR

Total RNA was extracted from mouse tissues or cell pellets using RNAiso (Takara Bio, Japan) followed by phenol-chloroform treatment. First-strand cDNA was synthesized from 1 µg of RNA using a cDNA synthesis kit (BioRad, USA) according to the manufacturer's protocol. The cDNA was then used for real-time PCR with SYBR Green Supermix (BioRad) on an Applied Biosystems Quant Studio™ 6 Flex Real-Time PCR System. Primer sets for gene detection are listed in Table S2.

Western blotting

Cells and mouse tissues were lysed using RIPA buffer (Sigma Aldrich) with 1X protease-phosphatase inhibitor (Thermo Scientific). Proteins separated by SDS-PAGE were transferred to a PVDF membrane and immunoblotted with primary antibodies against HIF-1α, IRF3, P-IRF3, and β-Actin (mentioned in Table S3). Secondary HRP-conjugated anti-mouse and anti-rabbit IgG antibodies (Table S3) were used for blot development. Standard protocol is described in our previous work[4,11].

Flow cytometry

PBMCs from the SARS-CoV-2 infected diabetic and non-diabetic individuals were treated with RBD peptide of SARS CoV-2 (Pep Mix SARS-CoV-2 S-RBD, Cambridge bioscience

Cat. No. PM-WCPV-S-RBD-1) for 24hr and stained with CD3 APC for 45 minutes. followed by fixation with fixation buffer (Invitrogen). further cells were permeabilized using permebilization buffer (Invitrogen) for 20 minutes followed by the intracellular staining with IFNγ PE. After staining for 2 hr cells were washed and analysed using a BD FACS Verse flow cytometer (BD Biosciences), and the data were processed with FlowJo software (FlowJo LLC, Oregon)[5,11].

ELISA

Maxisorp 96-well microplates (Thermo Fisher) were coated with fixed SARS-CoV-2 antigen at 1 μg/ml in PBS overnight at 4°C and then washed with PBS containing 0.1% Tween-20 (PBS-T). Plates were blocked with 2.5% bovine serum albumin in PBS-T for 2 hrs at room temperature. Plasma samples, diluted two-fold, were incubated with the antigen for 1 hr at room temperature and washed with PBS. Anti-mouse IgG HRP-conjugated (ImmunoTag) and anti-mouse IgM HRP-conjugated (Southern Biotech) secondary antibodies were added, incubated for 1 hour at room temperature, and washed with PBS. Plates were developed with TMB chromogen solution (Thermo Fisher), and the reaction was stopped with a stop solution (Thermo Fisher). Optical density was measured using a SpectraMax i3x multi-mode reader (Molecular Devices, USA). According to the manufacturer's instructions, IFN-α and IFN-β in mice were measured using ELISA kits (Elabscience).

CBA array

The CBA array was performed to measure cytokines IFN-γ (BDTM MS IFN-GMA CBA Flex set A4 100TST, BD) from plasma samples collected from SARS-infected mice of different treatment groups as described in the results and analyzed by CBA analysis software (BD Biosciences)[5].

Neutralization assay

Neutralizing antibody titers were calculated using a plaque reduction neutralization test (PRNT) assay. Serial dilutions of each mice plasma were incubated with 40-50 PFU of SARS-CoV-2 for 1 hr at 37°C. The virus-antibody immune complexes were transferred to the Vero E6 cell monolayer and plates were incubated for 2 hrs at 37°C in a 5% CO_2 incubator for virus adsorption. The CMC (Sigma) containing DMEM with 2% FBS and 1% penicillin-streptomycin was overlaid on infected Vero E6 cells monolayer and incubated for 2 days. After incubation cells were washed with PBS and fixed with 4% paraformaldehyde. Cells were then stained with 1% crystal violet for 10 minutes and washed with tap water. Plaques were counted. The 50% neutralization titer PRNT50 was calculated from non-linear regression curves in Graph Pad Prism version 10, as mentioned[4,5].

Hematoxylin and eosin staining:

The lungs of mice were excised and fixed in 4% PFA before being embedded in paraffin. Thin sections of 2.5 μm were cut and stained with hematoxylin and eosin (H&E). These slides were examined using a Nikon Eclipse Ti-E inverted stage microscope, and images were captured at 20× magnification. The cellularity score was then determined using ImageJ software[5].

RNA library preparation

For the transcriptomic study, RNA from the PBMCs was extracted using RNeasy Mini Kit (250 Qiagen), which followed the manufactured protocol. RNA samples were checked for the eligibility of the total RNA by using the Qubit RNA IQ Assay Kit in Qubit Flex Fluorometer from Applied Biosystem and High Sensitivity RNA ScreenTape Analysis in 4200 TapeStation

System from Agilent. rRNA from the cytoplasmic and mitochondrial source have been depleted to enrich the transcriptome. Integrated RNA was subjected to library preparation with a TruSeq Stranded Total RNA kit from Illumina. Following vendor's protocol single-strand cDNA was prepared after fragmentation using the reverse transcriptase enzyme. The process of adapter ligation was performed after the end repair and A-tailing of the double-stranded DNA. The library was enriched with a selected primer mix and 15 amplification cycles.

Next-generation sequencing and data analysis

The normalized library was pooled and a final concentration of 750pM of the pooled library was subjected for sequencing in P3 flow cell of the Illumina NextSeq 2000 platform. The sequencing was performed with 2X100 cycles of paired-end chemistry at Translational Health Science and Technology Institute (THSTI, Faridabad). The read quality of the raw data was evaluated using FASTQC, which flags any potential abnormalities that may have occurred during library preparation, or sequencing. (http://www.bioinformatics.babraham.ac.uk/projects/fastqc/).

The adapter and low-quality sequences were trimmed by Trimmomatic 0.39 (http://www.usadellab.org/cms/?page=trimmomatic). The pre-processed reads were aligned to the reference genome of *Homo sapiens* (GRCh38), using HiSat2 and assembled by StringTie2. The number of reads was counted in the alignment that overlapped with gene features using featureCounts 2.0.6. Within- and between-sample normalization was corrected by using full quantile normalization with the DESeq2 package from Bioconductor. The estimation of the differential expression gene profile (Fold changes) of all samples was calculated using DESeq2. Log 2 Fold changes 1 or 1.5 or 2 and P value less than 0.05 were considered up regulated, along with significant P value, and fold change less than -0.05 were considered down regulated. Network analysis was performed for significantly changed genes using clusterProfiler/bioMart R package, Networkanalyst, and ShinGO 8.0.

Generation of *HIF1A* knocked down cell lines

In the U937 monocytic cell line, the endogenous HIF1α expression was knocked down using liposome-mediated delivery (Thermo Fisher Scientific, USA) according to the manufacture's protocol. Opti-MEM media was used during transfection, followed by anti-biotic selection and knock down in cells was confirmed using Western blot assay, mentioned in S3I-J Fig and described in our previous work.[11]

Additional reference

11. Bhattacharya S, Shrimali NM, Mohammad G, et al. Gain-of-function Tibetan PHD2$^{D4E;C127S}$ variant suppresses monocyte function: A lesson in inflammatory response to inspired hypoxia. EBioMedicine. 2021;68:103418.
12. Agarwal S, Ghosh R, Verma G, Khadgawat R, Guchhait P* (2023) Alpha-ketoglutarate supplementation reduces inflammation and thrombosis in type 2 diabetes by suppressing leukocytes and platelet activation. *Clinical and Experimental Immunology*, https://doi.org/10.1093/cei/uxad086.
13. Dall'Agnese, A., Platt, J.M., Zheng, M.M. et al. The dynamic clustering of insulin receptor underlies its signaling and is disrupted in insulin resistance. Nat Comm 13, 7522 (2022). https://doi.org/10.1038/s41467-022-35176-7
14. Takemori H, Hamamoto A, Isogawa K, et al. Mouse model of metformin-induced diarrhea. BMJ Open Diab Res Care 2020;8:e000898. doi:10.1136/ bmjdrc-2019-000898
